# Editorial: Building the clinical research workforce: challenges, capacities and competencies

**DOI:** 10.3389/fphar.2024.1446908

**Published:** 2024-07-16

**Authors:** Carolynn T. Jones, Elizabeth Johnson, Barbara E. Bierer, Denise C. Snyder, Hazel Ann Smith, Emma Akuffo, Stephen Sonstein

**Affiliations:** ^1^ College of Nursing and Clinical Translational Science Institute, The Ohio State University, Columbus, OH, United States; ^2^ Mark and Robyn Jones College of Nursing, Montana State University, Bozeman, MT, United States; ^3^ Multi-Regional Clinical Trials Center of Brigham and Women’s Hospital and Harvard Medical School, Boston, MA, United States; ^4^ Division of Global Health Equity, Department of Medicine, Brigham and Women’s Hospital and Harvard Medical School, Boston, MA, United States; ^5^ Office of Clinical Research and Clinical and Translational Science Institute, School of Medicine, Duke University, Durham, NC, United States; ^6^ Staffordshire University, Stoke-on-Trent, United Kingdom; ^7^ Scientific Learning and Development Consultancy, London, United Kingdom

**Keywords:** clinical research competency, clinical research professionals, JTF framework, workforce development, retention

In this editorial, we summarize the identified headwinds evident in the clinical research professional workforce, ranging from capacity constraints to aligning competencies with the complexity of modern clinical research. This editorial is part of the Research Topic: “ *Building the Clinical Research Workforce: Challenges, Capacities and Competencies* ”. To move beyond common challenges, we outline opportunities for innovation in medical and pharmacological advancements from this Research Topic.

Over the past decade and especially in the past 5 years, there has been heightened attention to the available resources and training within the clinical research workforce. With pharmaceutical research sponsors spending an average of 50% more on research and development since 2018, and with much of this spending and investment in novel therapies coming from emerging biopharma companies, the criticality of a workforce pipeline cannot be overstated during periods of intense growth and market fluctuations in new drug and device development (Mullard, 2024). The foundation for core clinical research workforce competencies was established in 2014 with the initial publication of the harmonized Joint Task Force Clinical Trial Competency Framework (JTF Framework) to establish a common lexicon of critical workforce functional skills to adapt to innovative trial designs, complex trial conduct, and novel technologies (Sonstein et al.). By 2024, the framework, with translations in 11 languages, was being applied both in the United States and internationally to educate, train, and support the clinical research workforce (Joint Task Force for Clinical Trial Competency, 2017; Sonstein et al.). In the post-COVID-19 era, the aftershocks of increasing staff turnover rates and overall workforce contraction necessitated a harmonized response across a broad spectrum of employers: academic medical center research sites, cooperative groups, contract research organizations, and pharmaceutical companies, among others (Freel et al., 2023). The archetype of the clinical research professional (CRP) has expanded to include all individuals who support the operationalization of clinical research, including not only clinical research coordinators, clinical research nurses and midwives, but also advanced practice providers, pharmaceutical industry research physicians (e.g., medical monitors), regulatory affairs professionals, data management professionals, grant and contract administrators, ethics committee members, clinical laboratory personnel and managers, and quality assurance monitors and assistants (Mendell et al., 2024). This broad group of professionals continues to evolve but competency standards are necessary to meet the needs of a dynamic and constantly changing clinical research enterprise.

In addition to increasing staff turnover rates, additional challenges and gaps exist that affect institutions, researchers, and the CRP workforce. One gap is a generalized lack of public understanding of clinical research, which contributes to a lack of awareness that clinical research is a career path for future employees. The majority enter the profession “by accident” rather than having an intentional plan to enter the clinical research workforce at the end of secondary school (Freel et al., 2023) or higher education. As the general retirement cliff for the current CRP workforce approaches, attention is appropriately shifting to cultivating interest and inquiry among the next-generation of research-engaged graduates. This includes the opportunity to recruit and retain CRPs from diverse backgrounds and communities which in turn may facilitate a higher degree of relatability among members of the public and make them feel welcome to participate in research.

As part of an initiative to increase the integration of clinical research careers into higher education, a competency-based curriculum for training certificates, academic degrees, internships, and apprenticeships has been introduced to encourage earlier intentional entry into the field (Knapke et al.). (Kayla et al.) describe a workforce development and mentoring program specifically for research administrators, another group in the clinical research workforce experiencing staff retention challenges. The expanded adoption of decentralized or remote clinical trial models has challenged the enterprise to incorporate local talent sources, such as public health, home health, and community health workers to support the conduct of studies in non-traditional settings beyond academic medical centers and private practices (Besel et al.; Yakubov et al.). Research by (Besel et al.) provides insight into the needs of under-engaged populations, such as rural healthcare workers in cross-functional research that will enable optimal trial conduct and participant safety in variable healthcare resource settings. Additionally, the current CRP workforce lacks cultural diversity which can result in a downstream negative impact on participant recruitment for clinical trials (Tufts Center for the Study of Drug Development, 2021; Derk et al.). The inclusion of human resource departments and clinical research operational leaders in the creation of competency-based, standardized job titles, descriptions, and career progression has resulted in promising enhancements in the professionalism of these roles through better-defined upward mobility, professional development pathways and significantly reduced turnover (Snyder et al.).

The confluence of new talent pools and paradigmatic shifts in trial design has resulted in a refreshed JTF Framework that includes new emerging competencies to support its 8-domain structure, including project management competencies (Sonstein et al., 2022). Keim-Malpass et al. propose a curriculum model that focuses on dissemination and implementation (D&I) research methods and outcome assessment as important skills for researchers and CRPs. Multiple clinical research academic degree and training programs have embraced the JTF Framework as a curricular standard (Sonstein et al.) and a formal programmatic accreditation process is now available through the Commission on Accreditation of Allied Health Education Programs (2024). Process efficiencies in centralizing new hire JTF competency-based onboarding with on-demand online education (Cranfill et al.). Finally, digital badge micro-credentialing has been tested and is available for replication in other institutions and resource settings (Lee-Chavarria et al.).

Evaluating the impact of CRP onboarding, training and education programs on employee performance, satisfaction and retention can include a variety of performance metrics and interpretive feedback that permits the capture of the lived experience of CRP while navigating the new complexities of innovative trial designs and research outreach. Sundquist et al. used the JTF Framework to implement and evaluate training and performance metrics for the Canadian Cancer Center Network programs. The Competency Index for Clinical Research Professionals (CICRP) has been piloted as one of the many tools used to evaluate an academic education program in clinical research (Jones et al.) and multiple measures have been suggested for D&I evaluation (Keim-Malpass et al.). Evaluation should be considered early when implementing new CRP program initiatives. Evaluation may also include horizon-scanning to determine the influence of competency level as it pertains to managing the risks associated with the expansion of research portfolios or programs at institutions or facilities. (Besel et al.) compare the JTF competencies to categorical levels of risk associated with clinical research organizational leadership and departmental management competencies to identify flexible means of creating awareness of changes in risk (e.g., participant safety, regulations) as they relate to necessary training or expansion of institution-based educational initiatives. Risk-based models, while not new to clinical research, are becoming more prevalent in workforce readiness approaches particularly in healthcare systems that serve under- or never-engaged populations prioritized by federal law (e.g., the DEPICT Act, https://www.congress.gov/bill/117th-congress/house-bill/6584/text; the pediatric RACE Act, https://www.congress.gov/bill/115th-congress/house-bill/1231/text).

This collection reflects some of the recent trends and proposed solutions to recruit, educate and retain the current workforce while developing a strong talent pool for the future. The need for a sustainable clinical research workforce pervades global regions as adaptive, innovative trial designs and community emphasis on the co-creation of the research experience become commonplace requirements of regulatory and ethics committees. Prioritizing recruitment of populations historically under-engaged in clinical research requires a diverse research workforce and propagates the necessary inclusivity that has been elusive in past decades. In response, new competencies are emerging, and methods for evaluating outcomes and implementation at the individual and program levels are recommended at the start of projects to promote high levels of engagement and stakeholder buy-in during periods of intense change. This Research Topic of work embodies the commitment of researchers, institutions, and advocacy groups to ensure the advancement of novel therapies through dedicated clinical research professionals for decades to come.
